# Retinal vascular changes and aqueous humor cytokines changes after aflibercept intravitreal injection in treatment-naïve myopic choroidal neovascularization

**DOI:** 10.1038/s41598-018-33926-6

**Published:** 2018-10-23

**Authors:** Luca Di Antonio, Lisa Toto, Alessandra Mastropasqua, Lorenza Brescia, Emanuele Erroi, Alessia Lamolinara, Marta Di Nicola, Leonardo Mastropasqua

**Affiliations:** 1 Department of Medicine and Science of Ageing, Ophthalmology Clinic, University G. d’Annunzio Chieti-Pescara, Chieti, 66100 Italy; 2 Department of Medicine and Aging Science, CeSi-Met, University “G. d’Annunzio” Chieti-Pescara, Chieti, 66100 Italy; 3 Department of Medical, Oral and Biotechnological Sciences, Laboratory of Biostatistics, University “G. d’Annunzio” Chieti-Pescara, Chieti, 66100 Italy

## Abstract

The aim of the study was to assess retinal vascular changes using optical coherence tomography angiography (OCTA) and aqueous humour changes of vascular endothelial growth factor (VEGF) and placental growth factor (PIGF) levels in treatment-naïve myopic choroidal neovascularization (mCNV) after aflibercept intravitreal injection. To explore the correlation between clinical and laboratory parameters. Fifteen eyes of 15 patients with treatment-naïve mCNV underwent 2 intravitreal injections of aflibercept. Main outcome measures were best corrected visual acuity (BCVA), central retinal thickness (CRT) and external limiting membrane (ELM) visualization at OCT, lesion area and leakage at fluorescein angiography (FA), OCTA flow area and selected area at baseline and after the injections. Analysis of VEGF and PlGF in the aqueous humor was performed before each injection in cases and prior to cataract surgery on 10 patients as included as controls. Median BCVA increased from 0.6 to 0.3 logMAR (p < 0.001); CRT decreased from 387.5 to 267 micron (p < 0.001); FA area from 0.8 to 0.5 mm^2^ and OCTA area from 0.9 to 0.5 mm^2^ (p = 0.005). PIGF values changed from 1.8 to 1.4 pg/ml (p = 0.019) and VEGF values from 3.4 to 0.5 pg/ml (p = 0.008). A significant correlation was found after treatment between PIGF levels and BCVA (rho = 0.006) and VEGF levels and BCVA (rho = 0.018); between PlGF and CRT (rho = 0.020), PlGF and ELM visualization (rho = 0.002) and PlGF and FA leakage (rho < 0.001). Our results showed a significant reduction of mCNV area after aflibercept in both FA and OCTA measurements; an improvement of BCVA, and a reduction of VEGF and PIGF levels related to inactivity of the disease.

## Introduction

Pathological myopia (PM), also known as degenerative myopia, is a condition in which individuals have an axial length >26 mm corresponding to a refractive error of at least −6.0 diopters (D)^1^. The most frequent sight-threatening complication of PM is myopic choroidal neovascularization (mCNV), and nearly 10% of worldwide patients younger than 50 years with PM develop mCNV^2^. Fluorescein angiography (FA) has been used to detect mCNV and evaluate its shape, size and activity of the disease^3^. Spectral domain optical coherence tomography (SD-OCT) showed great sensitivity (about 97%) for detecting mCNV’s activity^4^. Recently a new, fast, safe and dyeless method, called optical coherence tomography angiography (OCTA) was been introduced into daily clinical practice. The OCTA gives more insight deep into the retinal and choroidal microcirculation by revealing the features of CNV that usually are not visualized with other imaging techniques^5^. Indeed OCTA is able to visualize the neovascular network of CNV better than FA, because it does not suffer of masking of dye. The OCTA examination demonstrated high sensitivity and specificity to detect mCNV^6^.

Previous therapies such as laser photocoagulation for extra/juxtafoveal CNV, and photodynamic therapy with verteporfin for subfoveal CNV have shown limited outcomes^7,8^. Anti-vascular endothelial growth factor (anti-VEGF) intravitreal injection has been recommended as first-line therapy for CNV-related PM, showing efficacy and safety as treatment strategy^9^.

Morphological changes of mCNV have been described after anti-VEGF drugs injections by means of multimodal imaging and ultimately using OCTA^10–12^.

In addition variations of aqueous humor cytokines after anti-VEGF drug treatment for mCNV have been described in several studies^10,13^.

The recent introduction of aflibercept (Eylea; Regeneron Pharmaceuticals, Tarrytown, NY, USA) for the treatment of mCNV provides an alternative mechanism of VEGF blockade by binding all isoform of VEGF-A, VEGF-B, and placental growth factor (PIGF).

In this study we assess retinal vascular changes by means of OCTA and aqueous humor VEGF and PlGF levels modifications after aflibercept intravitreal injection in treatment-naïve mCNV.

## Results

### Demographic data

Fifteen eyes (cases) of 15 patients (6 males and 9 females) with PM complicated by treatment-naïve mCNV and 15 eyes (controls) of 15 healthy age-matched subjects (5 males and 10 females) were included in this study (Table 1).

**Table 1:** Baseline characteristics of cases and controls expressed as median and interquartile range (Q_1_–Q_3_).

	Controls	mCNV	*p-value* ^a^
Age (yr), *mean* ± *SD*	63.1 ± 11.2	64.1 ± 12.1	*0.796*
Gender, *n(%)*			*0.567* ^b^
Male	5 (33.3)	6 (40.0)	
Female	10 (66.7)	9 (60.0)	
Refractive error (D)	−11.5 (8.75–13.25)	2.1 (1.2–2.2)	***0.005***
BCVA (logMAR)	0.3 (0.2–0.7)	0.6 (0.5–0.8)	*0.063*
CRT (µm)	206.0 (198.5–246.5)	387.5 (331.0–314.5)	**<** ***0.001***
PlGF (pg/ml)	3.0 (2.0–4.0)	1.8 (1.5–2.9)	***0.015***
VEGF (pg/ml)	13.5 (10.8–16.3)	3.4 (2.0–5.6)	**<** ***0.001***

BCVA, best corrected visual acuity; CRT, central retinal thickness; PlGF, placental growth factor; VEGF, vascular endothelial growth factor.

^a^Mann-Whitney U test *vs* controls; ^b^Chi Square test.

The mean age was 64.1 ± 12.1 years in cases and 63.1 ± 11.2 years in controls (p = 0.796).

Median and interquartile range of refractive error in diopters (D) was −11.5 (−8.75; −13.25) in cases and +2.1 (+1.2; +2.2) in controls (p = 0.005).

Morphological and functional parameters and aqueous level of cytokines were significantly different between cases and controls at baseline (Table 1).

### Functional and morphological results

BCVA significantly improved from T0 to T1 (p < 0.05 post hoc analysis) and did not change significantly at T2 (Table 2). Activity of the disease, evaluated by means of FA showed a significant reduction of both leakage and lesion area (p = 0.003 and p = 0.005, respectively) (Table 2) (Fig. 2).

**Table 2:** Median and interquartile range (Q_1_–Q_3_) of different parameters at different follow-up time in mCNV patients.

	T0	T1	T2	*p-value* ^a^
BCVA (logMAR)	0.6 (0.5–0.8)	0.3 (0.3–0.4)*	0.3 (0.3–0.4)	**<** ***0.001***
FA Leakage	2 (2–2)	—	0 (0–0.3)	***0.003*** ^**b**^
FA area (mm^2^)	0.8 (0.5–3)	—	0.5 (0.3–1.5)	***0.005*** ^**b**^
OCTA Selected Area (mm^2^)	0.9 (0.5–2.9)	0.6 (0.3–2)*	0.5 (0.2–1.5)	***0.005***
OCTA Flow Area (mm^2^)	0.5 (0.3–1.5)	0.3 (0.2–0.9)*	0.3 (0.1–0.6)	***0.013***
CRT (µm)	387.5 (331.0–435.8)	285.0 (257.5–333.5)*	267.0 (255.3–314.5)	**<** ***0.001***
ELM visualization	0 (0–0)	2 (1.7–2)*	2 (1.8–2)	**<** ***0.001***
PlGF (pg/ml)	1.8 (1.5–2.9)	1.4 (1.1–1.9)*	—	***0.019*** ^**b**^
VEGF (pg/ml)	3.4 (2–5.6)	0.5 (0.1–0.9)*	—	***0.008*** ^**b**^

BCVA, best corrected visual acuity; FA, fluorescein angiography; OCTA, optical coherence tomography angiography; CRT, central retinal thickness; ELM, external limiting membrane; PlGF, placental growth factor; VEGF, vascular endothelial growth factor.

^a^Friedman test; ^b^Wilcoxon U test.

*p < 0.05 post hoc analysis respect to previous follow-up control.

**Figure 1:**
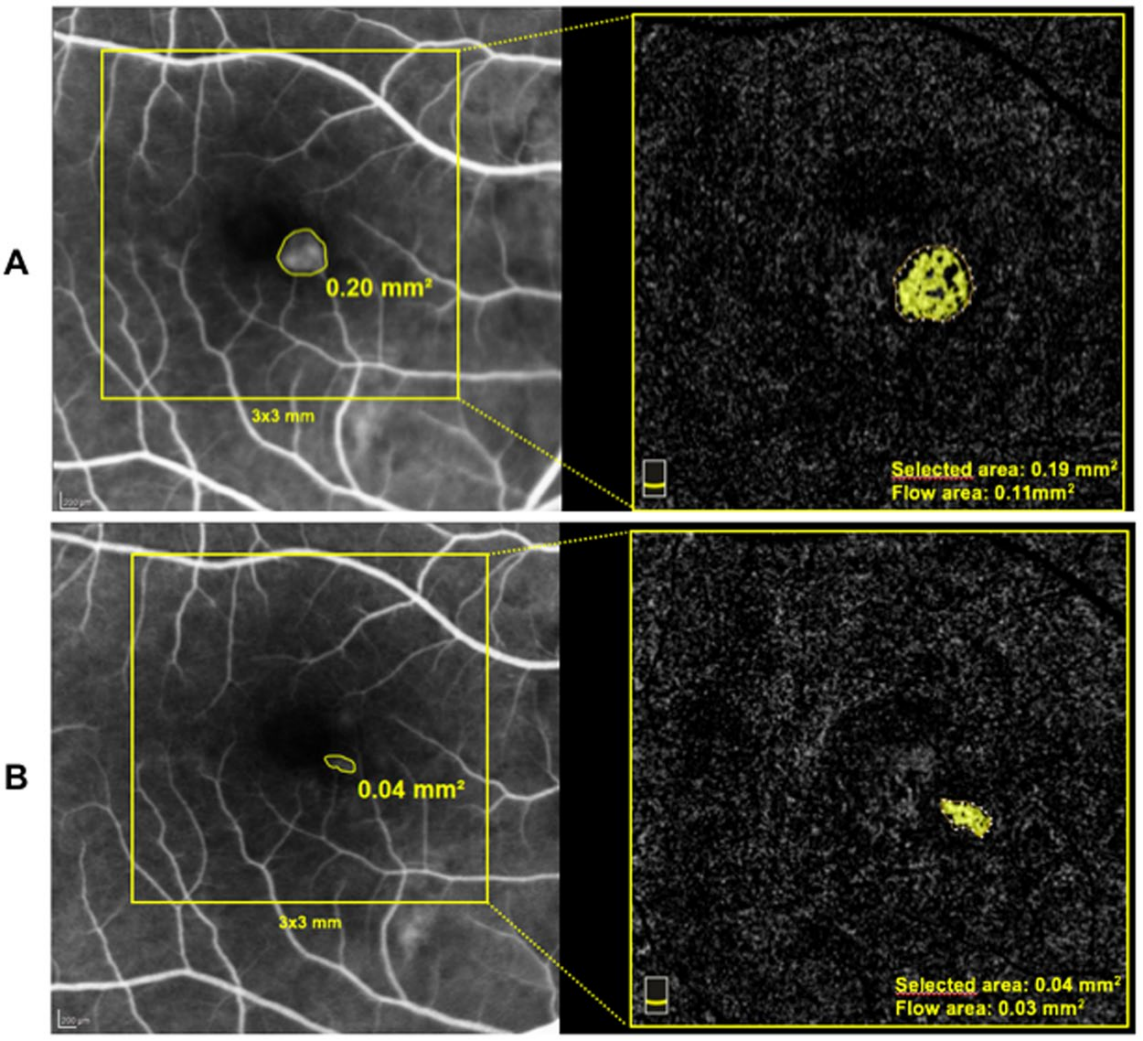
Fluorescein angiography and OCT angiography area measurements of mCNV at baseline (panel A) and after two aflibercept intravitreal injections (panel B). Note the agreement between FA and OCT angiography area measurements and the reduction of mCNV area after treatment.

Selected area and flow area at OCTA significantly decreased by two-fold at T1 (p < 0.05 post hoc analysis) and were almost stable at T2 (Table 2) (Fig. 1).

Macular thickness significantly reduced at T1 (p < 0.05 post hoc analysis) and was unchanged at T2 (Table 2).

ELM visualization was 2 (1.7–2) at T1 and was 2.0 (1.8–2) at T2 (Table 2).

### Cytokines quantitative analysis results

**Figure 2:**
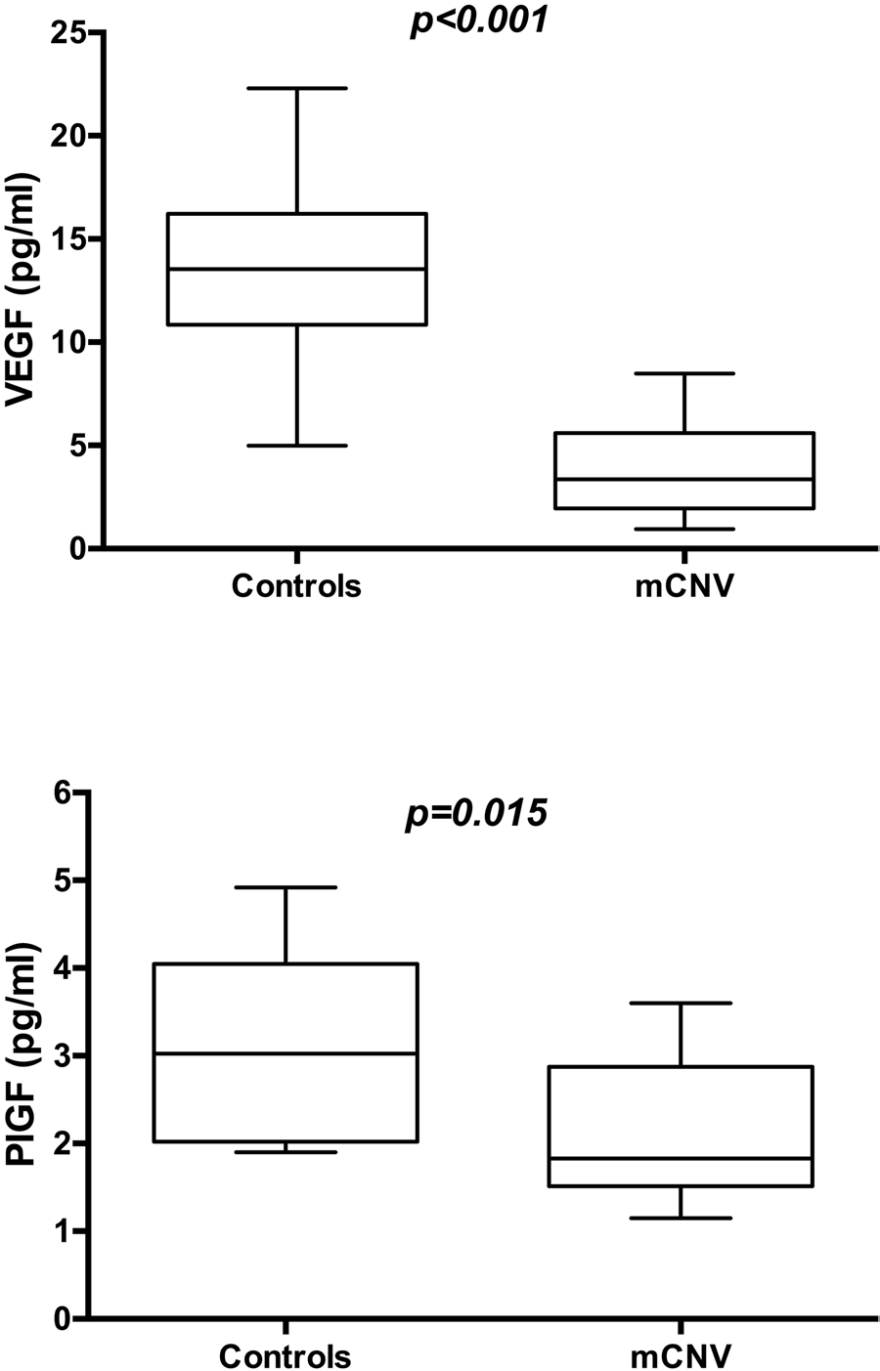
Box-whiskers graphs of VEGF (upper panel) and PIGF (lower panel) values in mCNV and control eyes. Box-whiskers plots show the 25th and 75th percentile range (box) with 95% confidence intervals (whiskers) and median values (transverse lines in the box). Mann-Whitney U test showed a statistical significant difference between two groups.

VEGF and PlGF were significantly different between cases and controls (Fig. 2) at baseline and in cases significantly decreased from T0 to T1 (p = 0.008 and p = 0.019, respectively) (Table 2).

### Correlations between clinical and laboratory variables

Before treatment there was a significant positive correlation between VEGF and BCVA (p = 0.013) and a significant negative correlation between VEGF and CRT (p = 0.001) (Table 3).

**Table 3:** Spearman’s rho correlation coefficient (p-value) between clinical parameter and VEGF and PIGF in mCNV patients at different follow-up times.

	T0		T1	
	VEGF	PlGF	VEGF	PlGF
BCVA (logMAR)	0.546 *(p* = *0.013)*	−0.230 *(p* = *0.221)*	0.525 *(p* = *0.018)*	0.595 *(p* = *0.006)*
CRT(µm)	−0.711 *(p* = *0.001)*	−0.219 *(p* = *0.245)*	−0.086 *(p* = *0.720)*	0.514 *(p* = *0.020)*
ELM visualization			−0.351 *(p* = *0.129)*	−0.659 *(p* = *0.002)*
FA Leakage			0.433 *(p* = *0.057)*	0.774 *(p* < *0.001)*
OCTA Selected Area	−0.030 *(p* = *0.899)*	0.442 *(p* = *0.051)*	−0.110 *(p* = *0.644)*	0.336 *(p* = *0.147)*
OCTA Flow Area	−0.030 *(p* = *0.899)*	0.442 *(p* = *0.051)*	0.031 *(p* = *0.898)*	0.269 *(p* = *0.251)*

Significant positive correlations were found between VEGF and PIGF and BCVA after treatment (p = 0.018 and p = 0.006, respectively) and between PIGF and CRT (p = 0.020) and PlGF and FA leakage (p < 0.001) (Table 3). A significant negative correlation was found between PIGF and ELM visualization (p = 0.002) (Table 3).

No significant correlations were found before and after treatment between VEGF and PlGF levels and OCTA parameters (Table 3).

## Discussion

Myopic CNV is the most frequent sight-threatening complication of PM. Although the gold standard treatment decision method is still based on FA late frames or on SD-OCT features, a novel dyeless technique for producing retinal imaging was widely speculated^2,3,5,6^.

Previous studies have demonstrated the ability of OCTA to characterize mCNV and have also reported high sensitivity of OCTA in detecting mCNV^6^.

Miyata *et al*. recently reported a 94.1% sensitivity of OCTA in myopic CNV detection with comparable area measurements in FA and OCTA, but they did not investigate CNV area measurements in both FA and OCTA after intravitreal therapy^6,11,12^.

Other authors showed a 90% sensitivity of OCTA for mCNV detection describing different pattern of mCNV^11,12^.

Cheng *et al*. and Cennamo *et al*. recently reported the use of OCTA as a useful tool to monitor vascular changes after anti-VEGF treatment. The authors did not use FA as a standard of care to assess the activity of the lesion, therefore they did not report a comparison between FA and OCTA of mCNV features^14,15^.

In our study we characterized findings of mCNV using OCTA before and after treatment with anti-VEGF agents and compared OCTA and FA features of mCNV. In addition aqueous humor VEGF and PlGF values before and after anti VEGF treatment were evaluated and correlated with functional and morphological parameters.

In our sample BCVA significantly improved after the first treatment and remained stable thereafter. Leakage and area of the mCNV at FA significantly decreased during follow-up. At SD-OCT examination macular thickness decreased and ELM visualization improved after the first intravitreal injection with no significant change following the second injection.

At OCTA examination both selected area and flow area significantly reduced with an almost two-fold decrease after the first treatment. These data are in agreement with those reported by Cheng *et al*. and Cennamo *et al*.^14,15^.

The usefulness of OCTA quantitative measurements in assessing the response of CNV to anti VEGF treatment has been demonstrated by other authors reporting reduction of CNV area at OCTA as a marker of responsiveness of the neovascular lesion to the treatment correlating to decrease of sub-retinal and intra-retinal fluid at SD-OCT^16^.

Our results confirm the usefulness of a no-invasive procedure such as OCTA in monitoring mCNV response to treatment with reduction of both selected and flow area during follow up.

Several studies show the potential role of VEGF and PlGF in the pathogenesis of neovascular and inflammatory conditions^17–19^. Cytokines quantitative analysis results showed a statistically significant difference between cases and controls at baseline with higher values of VEGF and PlGF in controls compared to cases. This finding is in accordance with previous report by Costagliola *et al*. that showed higher aqueous humor VEGF levels in normal subjects compared to mCNV patients^10^. The dilution effect of cytokines in the anterior chamber of myopic eyes has been suggested as the possible explanation^10^.

After treatment both aqueous humor VEGF and PlGF concentrations significantly decreased with greater reduction of VEGF levels (6.8 fold decrease) compared to PlGF (1.2 decrease).

Similar results were observed by Costagliola *et al*. that found a 3.9 fold reduction of VEGF after the treatment^10^. There is no report of PlGF changes in mCNV after intravitreal treatment.

Before treatment a significant correlation was found between VEGF and BCVA and VEGF and CRT (Rho = 0.546, p = 0.013 and Rho = −0.711, p = 0.001, respectively).

After treatment a significant correlation was found between VEGF and PIGF and BCVA (Rho = 0.525, p = 0.018 and Rho = 0.595, p = 0.006, respectively), between PIGF and CRT (Rho = 0.514, p = 0.020), between PIGF and ELM visualization (Rho = −0.659, p = 0.002) and between PIGF and FA leakage (Rho = −0.774, p < 0.001).

It is known that aflibercept is a fully human recombinant protein consisting of key binding domains from VEGF receptor (VEGFR)-1 and VEGFR-2 fused to an IgG Fc fragment and neutralizes all VEGF-A isoforms and inhibit also VEGF-B and PLGF-1 and -2^20^.

Our results suggest a role of both VEGF and PlGF in the development mCNV as intravitreal aflibercept treatment reduced CMT, improved FA and OCTA features and increased BCVA. In addition the significant correlations between VEGF and functional parameters and PlGF and morphological/functional parameters suggest an important role of these cytokines in mCNV pathogenesis. It is difficult to understand the different behaviour of the 2 cytokines investigated in our study, we can speculate that they contribute in different manner to the development of the mCNV and modify differently after treatment thus influencing in a different way the tissue response.

The significant correlation between PlGF and morphological and functional parameters after treatment suggest that this cytokine could be an important target for studying the therapeutic responsiveness of myopic new vessels.

The limits of this study are a relatively small sample size and short follow up. Future clinical trial with higher number of cases and longer follow up period are needed to better understand the role of these cytokines in mCNV pathogenesis and the usefulness of OCTA in patient monitoring and possible correlations between OCTA features and aqueous humor cytokines levels.

In conclusion our study demonstrated the usefulness of OCTA-based metrics in mCNV to quantitatively assess the response to the treatment with aflibercept showing the reduction of both flow and the size of CNV. Moreover ELM visualization and CMT by means SD-OCT and/or FA leakage are significantly related to VEGF and PlGF concentrations.

## Methods

In this prospective case-series study we enrolled only those patients with active treatment-naïve mCNV presented at the University Gabriele D’Annunzio Department of Ophthalmology, between December 2016 and August 2017 candidates to a fixed treatment regimen with 2 consecutive intravitreal injections of 2 mg aflibercept (Eylea, Regeneron Pharmaceuticals) 30 days apart from each other. The study adhered to the tenets of the Declaration of Helsinki and was approved by the Institutional Review Board of the Gabriele D’Annunzio University, Chieti, Italy. Informed consent was obtained before the enrollement. Inclusion criteria were age ≥18 years, best corrected visual acuity (BCVA) at least of 1.0 logMAR in the study eye, high myopia (defined by an axial length >26 mm or a refractive error of at least −6 D) and the presence of subfoveal or juxtafoveal (within 1–199 micron from the center of the fovea) active mCNV.

Activity of the mCNV had to be confirmed by both FA and SD-OCT, consisting by late leakage of the hyperfluorescent neovascular network and the presence of sub/or intraretinal fluid and/or subretinal hyperreflective exudation, respectively. Also the visibility of external limiting membrane (ELM) by means of SD-OCT was used as a parameter for evaluating CNV activity^21^. Patients with fibrotic mCNV or CNV attributable to any other causes than myopia, or subjects previous treated with other therapies were excluded from our study.

Each subject underwent a comprehensive ophthalmic examination, including BCVA using an Early Treatment Diabetic Retinopathy Study charts, slit-lamp biomicroscopy, intraocular pressure measurement with Goldmann applanation tonometry, dilated funduscopic examination, FA and SD-OCT using a confocal scanning laser ophthalmoscope (Spectralis HRA + OCT; Heidelberg Engineering, Heidelberg, Germany), and OCTA assessment by means of SD-OCTA (XR Avanti, Angiovue, Optovue Inc, Freemont, CA, USA) a commercially available device with a high-speed of 70,000 axial scans per second, using a light source of 840 nm, and an axial resolution of 5 μm. The AngioVue OCTA system is based on Split Spectrum Amplitude Decorrelation Angiography (SSADA) algorithm (version 2017.1.0.144). This algorithm identifies blood flow by calculating the decorrelation of signal amplitude from consecutive B-scans performed at the same retinal acquisition plane by splitting the spectrum, improving the signal-to-noise-ratio, and enhancing flow detection^22^. Study participants underwent OCTA imaging following a protocol that included two volumetric OCT dataset of 3 × 3 mm, consisting of 304 × 304 pixels in the transverse dimension. An internal fixation light was used to center the scanning area on the fovea. The image capturing time required about 3 seconds for each orthogonal volumetric scan by using an embedded eye tracking system (about 30 MHz). After completion of the volumetric OCT dataset, the software applied motion control technology to remove saccades and minor loss of fixation. Low-quality scans (i.e., if the subject blinked or the scan had significant motion artifacts) were excluded and repeated until good-quality scans were achieved. Automatic segmentation embedded in the machine was used to identify all (four) retinal layers as previously reported. In this study, in order to better identify the flow neovascular network of CNV we considered the outer retinal slab (from Bruch’s membrane to inner nuclear/outer plexiform layer junction) by using a projection artifacts removal (PAR) software embedded into the AngioVue device. The PAR software cleanly removes flow projection from avascular outer retinal layer while preserving the visibility of the other vascular layers.

When an intra/subretinal fluid and/or subretinal exudation were present, a slight adjustment from the clinician was necessary in order to avoid both image processing software and segmentation errors by using manual segmentation editing and propagation tool embedded into the AngioVue device. Briefly the user can manually correct one or a few affected B-scans and propagate the correction throughout the entire scan volume or through a user-selected region in order to obtain a better and appropriate definition of enface slab for qualitative and quantitative analysis, and for assessing change over time.

For CNV assessment FA, SD-OCT and OCTA measurements were considered.

We manually measured CNV area on the early frame of FA, using an area-measuring software provided by a multimodal scanning laser ophthalmoscopic (FA/SD-OCT) device, and two CNV area measurements: “select area” and “flow area” using a tool embedded in the OCTA instrument. Central retinal thickness (CRT) and ELM visualization assessment were evaluated by means SD-OCT.

The analysis of FA, SD-OCT and OCTA imaging measurements were reviewed by two retinal experts (LDA and LT). Intraclass correlation between the two readers was 0.99 (95% CI: 0.98–0.99) for selected area and 0.99 (95% CI: 0.98–0.99) for flow area on OCTA images.

Activity of the disease was established by means of leakage at FA and ELM visualization at SD-OCT. Leakage at FA was graded as 0 (not present), 1 (questionable), 2 (well defined). External limiting membrane visualization at SD-OCT was graded as 0 (not-visible), 1 (questionable) 2 (well defined).

In all patients the intravitreal injection of 2 mg (0.05 mL) of aflibercept was performed using the standard injection procedure in the theater room at baseline and about 1 month later. In each patient an aqueous sample was collected before each intravitreal injection by aspirating 0.05–0.1 mL of aqueous using a sterile syringe with a 30-gauge needle at the temporal limbus. Aqueous humor samples were rapidly frozen at −80 °C until assayed.

A control group of age-matched healthy patients undergoing cataract surgery was used for comparison of cytokines levels at baseline. Aqueous humour samples were obtained immediately before cataract surgery in the same fashion described for eyes with mCNV and were stored with same procedure.

### Multiplex analysis of samples

Samples were used to quantify the production of a cytokine and chemokine, VEGF, using Milliplex MAP Human Cytokine/Chemokine Magnetic Bead Panel-Immunology Multiplex Assay (HCYTMAG-60K, Millipore) and of an analyte, PlGF, using Milliplex Cardiovascular Disease Magnetic Bead Panel I (HCVD1MAG-67K, Millipore), according to manufacturer’ protocols. Briefly, undiluted aqueous humor samples (25 µl neat per well) were thawed and mixed well by vortexing prior to add to 25 μl of Assay Buffer. Then, 25 μl of magnetic beads, coated with specific antibodies, were added to this solution and incubated overnight at 4 °C with shaking. At the end of the incubation, the plate was washed twice with Wash Buffer and incubated 1 hour with 25 μl of biotinylated Detector Antibody at RT. Then, the plate was incubated for 30 minutes with Streptavidin–Phycoerythrin at RT, washed twice and incubated with 150 μl of Sheath Fluid for 5 minutes at RT. The plate was ran immediately on a Luminex® 100™/200™ platform (Luminex Corporation, Austin, TX) with xPONENT 3.1 software. Standard curves for each analyte (in duplicate) were generated using the reference standards supplied with the kit. Analytes concentrations in sample were determined with a 5-parameter logistic curve. Final concentrations were calculated from the mean fluorescence intensity and expressed in pg/mL. The assay was performed in a 96-well plate, using all the assay components provided in the kit. All incubation steps were performed in the dark to protect the beads from light.

### Main outcome measures

The main outcome measures were mCNV area in both FA and OCTA; activity of the disease (leakage at FA and ELM visualization at SD-OCT); CRT at SD-OCT and BCVA.

Best corrected visual acuity; CRT and ELM visualization and OCTA selected/flow area were measured at baseline (T0), 1 month (T1) and two months (T2). Fluorescein angiography area and FA leakage were measured at baseline (T0) and two months (T2).

In addition VEGF and PlGF concentration values at baseline and after one month were analysed.

### Statistical analysis

The quantitative variables were summarized as median and interquartile range (IQR) according to their distribution and qualitative variables as frequency and percentage. A Shapiro-Wilk’s test was performed to evaluate the departures from normal distribution for each variable.

Mann-Whitney U test was performed to evaluate differences in quantitative variables among control and mCNV patients.

Friedman test or Wilcoxon U test, when appropriate was applied to evaluate the statistical difference over the follow-up time. Post-hoc analysis, a priori defined, was applied with Dunn-Sidak method adjustment for multiple comparisons.

Spearman Rho correlation coefficients were performed to evaluate statistically significant correlation between morphological/functional parameters and quantitative analysis of aqueous humor cytokines. In all statistical tests the threshold of statistical significance will be assumed equal to p = 0.05. Statistical analysis were performed using IBM^®^ SPSS Statistics v 20.0 software (SPSS Inc, Chicago, Illinois, USA).
